# Diagnostic Value of Structural and Functional Neuroimaging in Autoimmune Epilepsy

**DOI:** 10.1155/2020/8894213

**Published:** 2020-12-14

**Authors:** Limei Luo, Nian Wei, Jing Wang, Yuemei Luo, Fei Yang, Zucai Xu

**Affiliations:** ^1^Department of Neurology, Affiliated Hospital of Zunyi Medical University, Zunyi, China; ^2^Prevention and Health Care, Affiliated Hospital of Zunyi Medical University, Zunyi, China; ^3^Key Laboratory of Brain Science, Zunyi Medical University, Zunyi, China

## Abstract

Epilepsy is a common nervous system disease, which affects about 70 million people all over the world. In 2017, the International League Against Epilepsy (ILAE) considered immune factors as its independent cause, and the concept of autoimmune epilepsy (AE) was widely accepted. Early diagnosis and timely treatment can effectively improve the prognosis of the disease. However, due to the diversity of clinical manifestations, the expensive cost of autoantibody detection, and the increased prevalence in Western China, the difficulty for clinicians in early diagnosis and treatment has increased. Fortunately, convenient and fast imaging examinations are expected to help even more. The imaging manifestations of AE patients were characteristic, especially the combined application of structural and functional neuroimaging, which improved the diagnostic value of imaging. In this paper, several common autoantibodies associated with AE and their structure and function changes in neuroimaging were reviewed to provide help for neurologists to achieve the goal of precision medicine.

## 1. Introduction

Precision medicine is increasingly important in modern clinical medicine, as it aims to obtain an early and accurate diagnosis and reduce the subsequent treatment failure and intervention in disease development, which usually involves a highly individualized patient management and multidisciplinary cooperation [1–3]. For clinicians, the goal of achieving precision medicine for autoimmune epilepsy is fraught with challenges.

Epilepsy is a chronic neurological disorder characterized by recurrent abnormal discharge of neurons. Its etiology is complex, and a lot of studies showed that autoimmune factors may participate in its occurrence and development [4–6]. In 2017, ILAE regards immune factors as one of its six independent causes, and more attention has been paid to the research progress of AE [7, 8]. The understanding of AE can be divided into two types: one covers all epilepsy related to systemic autoimmune diseases and the other mainly includes epilepsy related to nervous system autoantibodies [9, 10]. Here, we mainly focus on the latter. Early and accurate diagnosis of AE is important because affected patients have seizures that are resistant to common antiepileptic therapy but usually respond to immunotherapy [6, 11, 12]. Antibody testing has always been essential for the diagnosis and evaluation of autoimmune diseases. There are still some situations in the laboratory examination of AE, such as sensitivity/specificity of antibody testing, inconsistent antibody titer between serum and cerebrospinal fluid (CSF), and lack of the rare antibody test and low popularity [13–15]. It should be noted that there was no difference in prevalence or incidence between autoimmune and infectious factors for inflammatory lesions of the central nervous system, and more than 50% of patients do not have specific autoantibodies [16, 17]. In patients with presumptive autoimmune encephalitis, there was no significant difference in the clinical manifestations of antibody-negative cases and confirmed cases, and the therapeutic response to immunotherapy was similar [18]. Therefore, the better application of convenient and fast imaging examination cannot be postponed.

In recent years, many studies have found that there is a unique value in the diagnosis and prognosis evaluation of structural and functional neuroimaging features in patients with AE. These imaging technologies, including magnetic resonance imaging (MRI), functional MRI (fMRI), positron emission tomography (PET), and single-photon emission computed tomography (SPECT), have opened a new way for the diagnosis and treatment of diseases [19–22]. Although the imaging changes of AE are relatively new in the field of radiology, the aim of this paper is to review the major antibody subtypes and imaging changes of AE, provide a framework for radiologists to understand the relevant neuroimmunology, and help clinicians to identify the causes of epilepsy for early and precise treatment (Table 1).

## 2. Neuroimaging Techniques

The etiologies of epilepsy are varied and multifactorial in most cases. Autoantibody testing takes a long time and has not been carried out in some hospitals, but immunotherapy usually must be based on clinical presentation and quasiclinical results obtained during that time. And in these circumstances, the study of biomarkers may be further helpful for the early and accurate diagnosis of AE. In addition to CSF analysis, electroencephalogram, and physical examination, neuroimaging techniques are also included. Because of the special anatomy of the brain, here we focus on MRI, PET, and SPECT.

MRI is the most commonly used neuroimaging test for lesions of the brain parenchyma. The magnetic resonance signal is generated by the radiofrequency pulsations, and the selected pulse sequence will determine the appearance of the image. T1-weighted images have an advantage in the presentation of anatomic detail, but T2-weighted images are often needed to demonstrate pathology [23]. The purpose of MRI in epileptic patients includes etiology/differential diagnosis, follow-up observation, and preoperative evaluation [20]. Not only can T1 and T2-weighted be contrasted in conventional MRI images, fMRI can also improve the detection of pathological conditions [24, 25]. The fMRI is increasingly used to evaluate the relationship between brain activation and sensory/motor and cognitive activities, and its application in AE has also been reported [26–28]. The fMRI usually uses blood oxygen level-dependent (BOLD) contrast to locate brain function [29]. In this paper, fMRI not only refers to the application of BOLD technology but also includes magnetic resonance spectroscopy (MRS), diffusion tensor imaging (DTI), and so on.

PET is a nuclear medical imaging technology that can be used to investigate human metabolic processes. The 2-deoxy-2-18F-fluoro-*D*-glucose positron emission tomography/computer tomography (18F-FDG PET/CT) visualizes regional neuronal activity by measuring cerebral glucose, and 18F-FDG activity was projected to predefined surface pixels after stereotactic anatomic standardization [30]. In the field of inflammation and tumor, the use of FDG-PET imaging has been widely reported [31, 32]. In recent years, its application in AE is also on the rise.

SPECT is a high-resolution noninvasive imaging mode, which belongs to the category of functional imaging. The imaging principle is to image the gamma ray emitted from the patient's body and realize the imaging of body function and metabolism with the aid of single photon nuclide-labeled drugs [33, 34]. SPECT is not only widely used in the diagnosis and follow-up of cardiovascular diseases, tumors, and kidney diseases but also in epilepsy [35–38]. It can locate the active epileptic brain tissue according to the change of local cerebral blood flow and provide important basis in the preoperative evaluation of drug-resistant epilepsy [39, 40].

## 3. Structural and Functional Neuroimaging Imaging Features of AE

In the central nervous system (CNS), neural antigen-specific autoimmune diseases characterized by seizures and other symptoms have been identified. According to the existing reports, some patients with the CNS autoimmunity showed focal seizures alone or seizures as the most prominent clinical manifestation [41]. There is a tendency to classify autoimmune antibodies associated with central nervous system diseases into three categories: anti-NMDAR antibodies, limbic encephalitis- (LE-) related antibodies, and other antibodies [42]. They are organized as a review for those autoantibodies associated with AE and a description of the reported structural and functional imaging findings.

### 3.1. NMDAR Antibody-Related AE

The N-methyl-*D*-aspartate receptor (NMDAR) is a type of ligand-gated ion channel that mediates a major component of excitatory neurotransmission in the CNS, widely present in the brain. The anti-NMDAR encephalitis is the most common form of autoimmune antibody-mediated encephalitis, and the antibody caused a titer-dependent and reversible reduction of synaptic NMDAR through an approach of internalisation and crosslinking [43]. The specific binding of CSF antibodies to their cognate receptor leads to the functional decline and reversible reduction of NMDAR synaptic localization and surface density [44, 45]. For people with anti-NMDAR encephalitis, women are more likely to develop psychiatric disorders at the beginning, while male patients are more likely to have seizures initially [46, 47]. There are many types of epileptic seizures in NMDAR antibodies related to AE, including complex partial seizures, generalized tonic-clonic seizure, epilepticus state, and persistent intractable epilepsy, and some patients may have two or more types of seizures in the course of the disease [47, 48].

Although anti-NMDAR encephalitis has various symptoms and frequency of seizures, the MRI is usually normal in most cases. Previous studies have shown that the proportion of MRI abnormalities is less than 50%, with most of these abnormalities presenting T2 and fluid-attenuated inversion recovery (FLAIR) hyperintensity [49]. Lesions can be widely found in the cortex and subcortical white matter area; the most common is the temporal lobe, especially the medial temporal lobe (MTL), followed by the frontal lobe, and others include the thalamus, basal ganglia, cerebellum, and brainstem [50–53]. The only prognostic MRI finding in this type of encephalitis is progressive cerebellar atrophy. A long-term follow-up study found that some patients developed reversible diffuse cerebral atrophy and progressive cerebellar atrophy which is irreversible and is closely associated with a poor long-term prognosis [54]. Recurrence of encephalitis can be manifested as isolated atypical symptoms, suggesting involvements of the brainstem and cerebellum, but recurrence is not associated with abnormal MRI manifestations [55].

Some scholars confirmed that the functional connectivity of bilateral hippocampus decreased in resting state fMRI, and DTI revealed widespread changes in the white matter, especially in cingulate gyrus, which was related to the severity of the disease [28, 56]. In the brain functional activity analysis of 17 patients with anti-NMDAR encephalitis, the decrease of amplitude of low-frequency fluctuation values in the left precuneus, bilateral posterior cingulate gyrus, and cerebellum could be observed [57]. Leptomeningeal contrast enhancement was also observed in some patients [49]. A neurometabolic study showed that in a 31-year-old woman, MRS revealed a hypoglutamatergic state in the left prefrontal cortex, and the increased *N*-acetylaspartate (NAA) concentration was detected only in the left hemisphere with low metabolism [58]. The decrease of NAA concentration in the basal ganglia and thalamus was also observed on MRS, and the NAA signal returned to normal after the clinical symptoms subsided [22]. In a male patient who was admitted to hospital with epilepsy and was finally diagnosed with anti-NMDAR encephalitis, MRS indicated a decrease in NAA, and SPECT showed hyperperfusion in the right temporooccipital territory [59]. These findings are progressive reversibility with clinical improvement [60]. The SPECT in patient with anti-NMDAR encephalitis also indicated hyperperfusion in the basal ganglia and cortex, especially in the frontal cortex [56].

The correlation between FDG-PET findings and epileptic activity is always direct. Compared with MRI with poor sensitivity, FDG-PET showed more evidence in detecting the progressive stages of anti-NMDAR encephalitis [61]. The metabolic changes on FDG-PET vary widely and involve all the cerebral lobes, including the temporal and occipital lobes, insular cortex, basal ganglia, hippocampi, striatum, caudate nuclei, cerebellum, and brainstem [51, 62–64]. The FDG-PET images of anti-NMDAR encephalitis-associated epilepsy showed a pattern of decreased metabolism from the front to the back, that is, high metabolism in the frontal lobe, temporal lobe, and basal ganglia and low metabolism in the parietal occipital lobe, and the metabolic pattern could change with disease progression, treatment, and follow-up [63]. During the acute and subacute phases, antibody levels were high in all patients, and FDG-PET indicated severe hypermetabolism in the frontal, temporal cortex, and basal ganglia and hypometabolism in bilateral occipital lobes; in the early stage of recovery, diffuse cortical metabolism was the main feature, and the antibody levels of these patients were weak and positive at the same time; during the recovery period, abnormal metabolism and antibody levels returned to normal [61, 63, 65]. The comparison of FDG uptake in patients and healthy probands showed the cortical anteroposterior gradient and increased uptake in the striatum [64]. During the treatment, the deterioration of brain metabolism occurred when the clinical condition deteriorated, which was accompanied by severe extensive cortical hypometabolism and basal ganglia hypermetabolism [66]. Focal hypermetabolism of the left temporal lobe can be observed in the context of decreased diffuse cortical uptake, as the patient was in a long-term epileptic state throughout the course of the disease [61]. It is important to note that the manifestations of FDG-PET could be almost normal in patients without obvious clinical abnormalities and negative antibody recovery after treatment, but it may become abnormal again with the recurrence of the patient's condition; these abnormal manifestations include previous or new ones, and dynamic monitoring of FDG-PET showed a parallel relationship between cerebral glucose metabolism and clinical improvement [58, 61, 66].

### 3.2. Limbic Encephalitis-Related Antibodies

Autoimmune limbic encephalitis is an inflammatory disease of the central nervous system that mainly involves the MTL [67]. Although the types and techniques of antibody testing have improved, there are still a number of patients who are antibody-negative and can get a certain degree of clinical improvement by immunotherapy [68]. In the absence of antibody test results or negative antibody detection, LE can be diagnosed by clinical symptoms and abnormal T2/FLAIR high signal intensity of bilateral brain parenchyma highly limited to MTL on MRI [69]. The related autoimmune antibodies mainly include AMPAR, LGI1, and GABABR [42].

#### 3.2.1. AMPAR Antibody-Related AE

The *α*-amino-3-hydroxy-5-methyl-4-isoxazolepropionic acid receptor (AMPAR) is a type of excitatory ionotropic glutamatergic receptor, which participated in majority of rapid excitatory synaptic transmission activities in the brain. Anti-AMPAR encephalitis patients contain antibodies against GluR1/2, which changes synaptic localization and number of AMPARs [70]. Anti-AMPAR selectively eliminated the surface and synaptic AMPARs and caused the amplitude and frequency of the microexcitatory postsynaptic current in neurons induced, resulting in the steady-state decrease of inhibitory synaptic transmission and the enhancement of intrinsic excitability, which may be an important cause of memory impairment and epilepsy [71, 72].

AMPAR antibody-associated encephalitis is relatively rare in AE and is often associated with MTL abnormals on MRI [73, 74]. In a 66-year-old woman with anti-AMPAR encephalitis and diabetes mellitus, MRI showed only a few high-intensity punctate lesions in the white matter, but FDG-PET revealed a wide range of low metabolism areas including the frontal, parietal, and temporal parahippocampal areas [65]. Anti-AMPAR encephalitis with generalized seizures was closely associated with sustained hypermetabolism of left hippocampal FDG in FDG-PET [75]. In a pregnant woman with anti-AMPAR encephalitis, the initial brain MRI showed bilateral marginal encephalitis, but with clinical progression, rapid brain atrophy appeared on MRI, and extensive cortical cortex, caudate metabolism, and brain stem perfusion were observed on FDG-PET [74]. Analysis of FDG-PET was performed in 2 patients with anti-AMPAR encephalitis without seizures. One patient presented with bilateral cerebellar hypermetabolism and the other with total cortical hypometabolism [76]. The metabolism of FDG in the brain is correlated with clinical manifestations, and abnormal metabolism turned to normal after antiepileptic and immune treatment [65, 75]. Due to the low incidence of anti-AMPAR encephalitis, current functional imaging studies are limited to PET imaging, and further SPECT and fMRI studies are needed.

#### 3.2.2. GABABR Antibody-Related AE

Gamma-aminobutyric acid (GABA) is an inhibitory neurotransmitter that exists in the CNS, which can decrease the excitability of neurons and plays a significant role in the regulation of muscle tone. The GABABR is a G protein-coupled receptor composed of two subunits, GABA-B1 and GABA-B2, both of which are essential for the receptor to perform its functions [77]. GABABR antibodies bind to the extracellular domain of the GABA-B1 subunit, which is an inhibitory receptor associated with seizure and memory dysfunction when disrupted [78]. GABABR antibody-associated encephalitis is characterized by epilepsy and is associated with other conditions such as opsoclonus-myoclonus syndrome, ataxia, and small-cell lung cancer [78, 79].

MRI findings in most cases of GABABR encephalities showed T2/FLAIR hyperintensity in medial temporal lobes [73, 78, 80]. Atrophy and hypointensity of the MTL were also found in rare cases [81]. In a prospective study of 15 patients, all patients developed seizures, and in 13 patients, the seizures were the presenting symptom. MRI indicated MTL T2/FLAIR hyperintensity in 10 patients, increased FLAIR signal in the corpus callosum in 1 patient, and normal in 4 patients [78]. MRI findings can reflect the progress of the disease to a certain extent. The involvement of the limbic system in the group with poor prognosis is more extensive than that in the group with good prognosis [80]. MTL hypermetabolism is the most common manifestation in FDG-PET [82]. A 55-year-old male presented with progressive seizures at 3 weeks with a high anti-GABABR antibody titer in CSF. FDG-PET showed significant MTL hypermetabolism and hypometabolism in other parts of the brain, but there were no related abnormal findings on MRI [83]. SPECT revealed that the hypoperfusion areas were consistent with the high expression area of GABABR, and the uptake of the motor area and left temporal lobe was increased, which may be related to convulsive seizures and tongue movement disorder, and all areas showed normal absorption following corticosteroid treatment and neurologic improvement [84]. Anti-GABABR encephalitis in MRS also suggested inflammatory changes, mainly manifested as decreased NAA and elevated lactate peaks [85].

#### 3.2.3. LGI1 Antibody-Related AE

Leucine-rich glioma-inactivated 1 (LGI1) is a secreted neuronal protein that interacts with voltage-gated potassium channels Kv1.1 to perform its functions. The patient's antibodies destroy the LGI1 signal transduction around synapses, leading to neuronal overexcitement and reduced plasticity [86]. Anti-LGI1 encephalitis is the second most common type of autoimmune encephalitis known, which can lead to memory impairment and various forms of seizures, among which faciobrachial dystonic seizure (FBDS) was representative to a certain extent [87, 88]. LGI1 gene mutation is associated with autosomal dominant temporal lobe epilepsy that seizures can be well controlled by antiepileptic treatment [89].

MRI abnormalities in MTL at the early stage of the disease are an important basis for the diagnosis of anti-LGI1 encephalitis [90, 91]. MRI abnormalities in anti-LGI1 encephalitis are most common in MTL and basal ganglia with T2/FLAIR hyperintensity [90, 92–95]. Other manifestations may also involve extratemporal structures which include insula, thalamus, and frontal cortex; however, cortical involvement beyond the limbic region on MRI is rare [90, 94–96]. MRI findings are abnormal in patients with FBDS, usually located in the basal ganglia, in which T1 hyperintensity can be a useful biomarker for FBDS [97, 98]. Radiologic progression was also noted. Most patients showed T2/FLAIR hyperintensities on conventional MRI in the hippocampus during the acute phase of the disease [91]. Changes in MTL and hippocampal volume from swelling to atrophy were observed during the follow-up, and anti-LGI1 encephalitis can be considered as a potential cause of MTL sclerosis [92, 99, 100].

The results of the fMRI study on 27 sufferers with anti-LGI1 encephalitis showed that the disease had extensive damage to brain network connections, including the change of the brain default mode network, and it suggested that the hippocampal damage and the increase of brain default mode network connections might be a compensation mechanism for memory damage [91].

The PET-CT obtained in the acute disease stage often showed FDG hypermetabolism in the affected area. In a study of 18 anti-LGI1 encephalitis patients with seizures, abnormalities were found in 50% of patients and most commonly involved the middle temporal lobe [94]. Even if MRI indicated no structural changes in the brain, abnormal FDG uptake could be seen on PET [101]. The hypermetabolism of bilateral temporal lobes shown in the PET-CT of the studied patients corresponded to the patient's seizure pattern [99]. Hypermetabolism in the striatum and cerebellum was also observed [52, 102]. There was a significant correlation between anti-LGI1 encephalitis with FDBS and basal ganglia [100]. In a study, five out of the eight patients had hypermetabolic abnormalities in basal ganglia [101]. One case of anti LGI1 encephalitis complicated with FDBs showed hyperintense T1 signal in basal ganglia on MRI, while hypermetabolism was found in the same area on PET [98]. However, the location of the MRI results is not always consistent with that of the FDG-PET, and this is a hint that the LGI1 antibodies may affect sugar metabolism and the hippocampus structure through two different steps [94].

### 3.3. Other Antibodies

#### 3.3.1. GABAAR Antibody-Related AE

GABAARs are a class of ligand-gated ion channels, and its main epitope targets were the *α*1/*β*3 subunits of the GABAAR [103, 104]. The antibodies caused a decrease in synaptic GABAAR selectivity, and high antibody titers in CSF and serum are associated with brain parenchymal lesions with seizures and/or intractable status epilepticus [105]. Compared with adults, children were more likely to have generalized seizures in GABAAR antibody-associated encephalitis; this disorder is severe, but most patients respond to treatment [104].

MRI abnormalities in most cases of anti-GABAAR encephalities showed not only multifocal cortical-subcortical T2/FLAIR abnormalities and predominantly involved temporal and frontal lobes but also basal ganglia and other regions [104, 106]. Epileptic persistence accompanied with extensive cortical-subcortical MRI abnormalities and limbic involvement occurs and is often accompanied by stiff-person syndrome [105, 106]. MRI pathologies are associated with disease progression and can be resolved completely after early immunoregulatory therapy [106]. In febrile infection-associated epileptic syndrome caused by GABAAR antibody with refractory status epilepticus, MRI remains consistently negative over the course of the disease, despite the epileptic discharge shown by electroencephalogram [107]. In a 67-year-old woman with anti-GABAaR encephalitis, MRI demonstrated a multifocal cortical-subcortical lesion, and MRS showed elevated lactate signals and Lac/creatine ratio in the voxel of interest [108].

#### 3.3.2. CASPR2 Antibody-Related AE

Contactin-associated protein 2 (CASPR2) is a transmembrane axonal protein localized at the juxtaparanodes of myelinated axons, a specialized region between axons and myelinating glial cells, and contributes to the jump conduction of action potential [109, 110]. CASPR2 autoantibodies are predominantly IgG4, which target multiple epitopes on the extracellular domain of the protein and destroy the combination of CASPR2 with contactin-2; it may interfere with the accumulation of of voltage-gated potassium channel (VGKC) at juxtaparanodes and leading to hyperexcitability of peripheral nerves [110, 111]. CASPR2 antibodies can also bind to hippocampal inhibitory interneurons at the presynaptic level and have a disruptive effect on inhibitory synapses [112]. Seizure is the first symptom, and sometimes, it is the only clinical manifestation in some CASPR2 antibodies positive patients; immunotherapy has a good effect on clinical improvement [113]. Patients can have a variety of seizures, including generalized tonic-clonic, which is the most common form of seizures, and the rest also includes simple partial seizures, complex partial seizures, and even epileptic persistence [113, 114].

MRI is a highly sensitive and specific predictor for CASPR2 encephalitis [115]. MRI abnormalities in anti-CASPR2 encephalitis are most common in MTL with T2/FLAIR hyperintensity [114, 116, 117]. Such abnormal changes in T2 can be restored to normal after immunotherapy [115]. Bilateral hippocampal atrophy was observed in the first MRI analysis in 13.3% of patients [114]. Generalized cortical atrophy and diffuse meningeal enhancement were also found in a small number of patients [113, 117].

In a retrospective study, FDG-PET was performed in 35 patients with CASPR2 autoantibodies-related diseases, of which 85.7% was abnormal, which commonly included temporomandibular hypometabolism (36.7%), frontal hypometabolism (20%), temporal hypermetabolism (16.7%), and diffuse hypometabolism (10%) [118]. FDG-PET showed reduced FDG uptake in one case of anti-CASPR2 encephalitis with generalized seizures, especially in orbitofrontal regions bilaterally, as well as in bilateral anterolateral temporal and left medial temporal regions [119]. A 72-year-old man with positive CASPR2 antibodies presented with hallucinations and seizures, on FDG-PET, revealed hypometabolism in the left temporal and occipital cortex [120]. The imaging findings of FDG-PET in two patients with anti-CASPR2 encephalitis were studied retrospectively: one patient had hypometabolism in association cortices and hypermetabolism of striata and the other showed normal [52]. There are no SPECT and fMRI studies on CASPR2 antibodies associated with AE, and further functional imaging studies are needed.

#### 3.3.3. GAD Antibody-Related AE

Glutamic acid decarboxylase (GAD) is an intracellular enzyme expressed in GABAergic neurons, which catalyzes the transformation of glutamate into GABA [121, 122]. GAD antibodies interfere with the endocytosis of GABAergic neuron vesicles and have been proved to be related to immune response [123, 124]. Acute seizures and chronic epilepsy with temporal lobe onset have been reported in patients with GAD encephalitis [125, 126]. Seizure can be the only clinical symptom of it [127]. A study on the etiologies of temporal lobe epilepsy shows that GAD antibodies were positive in 21.7% of the unknown etiology group, and epilepsy in patients with high antibody titers is often drug-resistant and has been linked to depression, memory disorders, and other autoimmune diseases [128].

The MRI manifestations of GAD antibodies associated to encephalitis involve a wide range, including the thalamus, insulae, parietal lobe, and brain stem, in addition to the most common temporal lobes [127, 129]. Patients in the acute/subacute setting often present with temporal encephalitis evidenced by T2/FLAIR hyperintensity and swelling of temporal structures [127, 130]. Hippocampal atrophy has been found in patients with GAD positive drug-resistant temporal lobe epilepsy [131]. Compared with the patients with positive anti-NMDAR and anti-VGKC antibodies, anti-GAD encephalitis showed higher FLAIR intensity in hippocampus on postprocessed images [130]. A volumetric analysis of serial MRIs indicated that the amygdala volume was increased obviously within the first 12 months after the onset of GAD encephalitis and and tended to be normal during the follow-up period; the increase of hippocampal volume showed no significant difference from the control group [132]. It is worth noting that although early immunotherapy is helpful to avoid brain injury, MRI abnormalities may not be visible in patients with anti-GAD in the early stages of acute immunoactivation [133].

DTI was used to study the changes of white matter in anti-GAD encephalitis, and the results showed that there was a wide range of effects in various regions of the brain, including wide changes of fractional anisotropy and all diffusivity parameters, and lesions with a trend toward a negative correlation of figural memory performance with diffusivity parameters were mainly appeared in the right temporal lobe [134].

The relevant FDG-PET results showed that high metabolism corresponds to early swelling of the lesion parenchyma, and low metabolism corresponds to atrophy at the later stage of the lesion process [127]. In patients with anti-GAD encephalitis, FDG-PET showed multiple hypermetabolism in brain tissue, mainly in the frontal or temporal lobes [127, 135]. When patient presents with cognitive decline, FDG-PET indicated bifrontal hypometabolism and hypoperfusion [136].

#### 3.3.4. Anti-Hu Antibody-Related AE

Anti-Hu antibody is a kind of antinuclear antibody that is related to a variety of tumors, including neuroendocrine tumor of the duodenum, neuroblastoma, and small cell lung cancer (SCLC) [137–139]. The paraneoplastic neurological syndromes associated to anti-Hu are severe and have no effective treatment. Its pathogenicity is believed to be related to nerve cells death and T cell immune response [140, 141]. Epilepsia partialis continua and intractable epilepsy are associated with Hu-ab [138, 142, 143]. The management of epilepsy was difficult in those epileptics without cancer-received antiepileptic drugs and immunotherapy [144].

When epilepsy occurs in patients with anti-Hu encephalitis, the most common abnormality on MRI was T2/FLAIR hyperintensity in the temporal lobe [19, 138, 142, 144]. In a boy with anti-Hu encephalitis, his MRI had no abnormality at the time of paroxysmal ataxia at first, but T2/FLAIR hyperintensity in the temporal lobe appeared after intractable epilepsy, and this change was consistent with electroencephalogram [142]. In patients' combination with SCLC, MRI showed multifocal subcortical/subcortical lesions with T2/FLAIR hyperintensity without any contrast enhancement in T1-weighted images, and with the development of the disease, brain atrophy and ventricular enlargement may occur [145]. Abnormalities in T2 may represent the sequelae of recurrent seizures, and changes from focal to multifocal may be observed in the course of the disease [146].

In the case of paraneoplastic limbic encephalitis, FDG-PET usually shows high metabolism in one or two temporal lobes, but only a small number of brain MRI cases are related to FDG-PET [147]. FDG-PET is particularly useful for diagnosis, recurrence, and evolution of tumors for anti-Hu-related AE [145, 147]. When patients with anti-Hu paraneoplastic syndrome developed partial status epilepticus, SPECT scan revealed asymmetric cortical activity, but could not identify obvious epileptic foci [148].

There are also some autoantibodies related to AE, such as those related to recombinant dipeptidyl peptidase 6, Ma2, mGluR5, and so on [7]. However, there are few reports or no specific manifestations on neuroimaging; so, we will not list them in this review.

## 4. Conclusions and Future Perspectives

The current diagnosis of AE relies too much on antibody detection and immunotherapy response. However, many institutions are not easy to carry out antibody detection, and it takes some time to obtain the test results, and it is not easy to obtain the information of immunotherapy response in the early stage. When the status of autoantibodies is not clear, clinical syndrome and imaging findings can determine the diagnosis of probable or definite autoimmune encephalitis [69]. Therefore, the value of convenient imaging in the diagnosis of AE should be paid more attention. MRI and PET are important imaging methods for detecting parenchymal lesions, and they have their own advantages and disadvantages. MRI is more readily available and is essential for preoperative evaluation of epileptic lesion resection [20]. Due to the wide popularity of magnetic resonance, many large sample data can be obtained, and sometimes, PET does not seem particularly important. One study showed that anti-LGI1 LE affects a wide range of brain regions, including the medial temporal lobe and basal ganglia, and these changes can be detected by head MRI without the need for PET/CT [96]. Multiple studies have found that PET is more sensitive than MRI because it can be abnormal in patients with normal MRI, and it is a trend toward that PET could be better used as an early biomarker for AE, so that treatment can start in the early phase [30]. Region-specific changes in brain FDG uptake occurred throughout childhood; so, age-specific adjustments were necessary in the statistical analysis of studies comparing FDG images of children's brains [149]. Considering the characteristics of the two technologies, simultaneous PET/MRI combines metabolic information of PET to localize the abnormality with high-resolution structural and functional information of MRI and holds the dual advantage of providing PET and MRI in single temporal as well as spatial domain [150].

At present, there are still some deficiencies in the research of functional imaging. On the one hand, there is a lack of large-scale prospective research on the causal relationship between brain dysfunction and autoimmune epilepsy. On the other hand, for some antibody types, due to the lack of functional imaging data, AE related to AMPAR, GABAAR, and CASPR antibodies, there is still a lack of SPECT- and fMRI-related research, so specific brain functional imaging changes cannot be obtained. Therefore, further large-scale clinical imaging research is needed in the future.

Precision medicine is extremely important in modern clinical medicine. It is an ideal goal that involves early accurate diagnosis of disease and customizes the optimal treatment plan. Because delayed immunotherapy is associated with poorer prognosis and higher mortality, the diagnosis of AE requires consideration of multiple factors. Antibody status as the only criterion for early diagnosis is clearly unrealistic. Convenient and fast neuroimaging can be used as an essential reference index for the diagnosis of AE. Both structural and functional neuroimaging techniques are particularly important in diagnosing and assessing disease progression. According to the current research, there is a tendency to combine the two to make better clinical decisions.

## Figures and Tables

**Table 1:** Types and imaging characteristics of antibodies related to AE.

Antibody types	MRI		PET	SPECT
	Regular MRI	fMRI		
NMDAR antibody	T2/FLAIR hyperintensity in the cortex and subcortical white matter areas, including temporal lobe, cerebellum, thalamus, basal ganglia, etc.	Bilateral functional connectivity of hippocampus decreased. DTI revealed widespread changes in white matter. The decrease of NAA is related to clinical improvement	A high to low metabolic gradient from the frontal lobe to the occipital lobe	Hyperperfusion in basal ganglia and cortex, especially frontal cortex

Limbic encephalitis-related antibodies	T2/FLAIR hyperintensity in MTL. MTL and hippocampal volume from swelling to atrophy	Extensive damage to brain network connections. MRS showed that NAA decreased and lactate peak increased	MTL hypermetabolisma is the most common manifestation	Hypoperfusio-n in the frontal lobe, parietal lobe, thalamus, and cerebellum

GABAAR antibody	Multifocal cortical-subcortical T2/FLAIR abnormalities, predominantly involved temporal and frontal lobes but also basal ganglia and other regions	MRS showed elevated lactate signals and Lac/creatine ratio in the voxel of interest	—	—

CASPR2 antibody	T2/FLAIR hyperintensity in MTL and diffuse meningeal enhancement. Bilateral hippocampal and generalized cortical atrophy	—	Temporal hypermetabolism, temporomandibular, frontal and diffuse hypometabolism	—

GAD antibody	Acute/subacute lesions usually presented as temporal lobe encephalitis with high T2/FLAIR signal and swelling of unilateral or bilateral medial temporal structures. Hippocampal atrophy is associated with drug-resistant temporal lobe epilepsy	DTI showed wide range of effects in various regions of brain	Multiple hypermetabolism in brain tissue, mainly in the frontal or temporal lobes	—

Anti-Hu antibody	The most common abnormality on MRI was T2/FLAIR hyperintensity in the temporal lobe and showed multifocal subcortical/subcortical lesions in patients with SCLC	—	High metabolism in one or two temporal lobes, only a small number of brain MRI cases are related to PET	SPECT scan revealed asymmetric cortical activity, but distinct seizure focus could not be identified
